# DEP regimen for the treatment of hemophagocytic lymphohistiocytosis: a review of published experience

**DOI:** 10.3389/fphar.2025.1599873

**Published:** 2025-06-11

**Authors:** Guangqiang Meng, Saran Feng, Yan Wang

**Affiliations:** Department of Hematology, The First Affiliated Hospital of Shandong First Medical University and Shandong Provincial Qianfoshan Hospital, Jinan, China

**Keywords:** hemophagocytic lymphohistiocytosis, DEP regimen, salvage treatment, induction treatment, treatment response

## Abstract

Hemophagocytic lymphohistiocytosis (HLH) is a life-threatening hyper-inflammatory clinical syndrome characterized by a storm of inflammatory factors. In the treatment of HLH, it is critical to provide active and effective treatment immediately and hamper the inflammatory cytokine storm in a timely manner to improve patient symptoms. Currently, the first-line treatment for HLH is still based on etoposide and glucocorticoids. Unfortunately, the treatment effect of HLH remains insufficient, the mortality rate of patients remains high, and the prognosis remains poor. Therefore, effective salvage treatments are urgently needed to alleviate relapsed and refractory HLH. More than 10 years have passed since the liposomal doxorubicin combined with etoposide and methylprednisolone (DEP) regimen was first reported as a salvage treatment for HLH. In more than 10 years of clinical practice, many studies have reported the effectiveness and safety of the DEP regimen for the treatment of HLH. The DEP regimen not only demonstrated a good salvage treatment effect in relapsed refractory HLH but also revealed an optimal therapeutic effect in first-line induction treatment of HLH.

## Introduction

Hemophagocytic lymphohistiocytosis (HLH) is an immune-mediated inflammatory disease characterized by high inflammatory factors (Ponnatt et al., 2022). The typical clinical manifestations of HLH include fever, hemocytopenia, hepatosplenomegaly, and other symptoms. HLH treatment mainly involves controlling the storm of inflammatory factors and clearing the primary cause of the disease; however, the overall treatment effect is poor (Griffin et al., 2020; Jordan et al., 2011). For example, approximately 25%–50% of patients with HLH do not achieve remission with regimens that include etoposide, dexamethasone, or methylprednisolone (Marsh et al., 2017). The liposomal doxorubicin combined with etoposide and methylprednisolone (DEP) regimen has been proven to provide good therapeutic effects in different types of HLH treatments.

### DEP regimen for salvage treatment of HLH

In 2014, Wang et al. first reported a clinical study on the application of DEP regimen for the treatment of refractory HLH in adults (Wang et al., 2014). The researchers administered salvage treatment to 41 patients who did not achieve partial response (PR) after 2 weeks of treatment with HLH-94. The results showed that among refractory patients, the complete response (CR) rate after treatment was 29.3%, the partial response rate was 48.8%, and the overall response rate (ORR) was 78.1%. The results of this study showed that the DEP regimen was effective in saving patients with refractory HLH who responded poorly to the HLH-94 regimen. Subsequently, Wang et al. reported a multicenter clinical study of a DEP regimen for the salvage treatment of refractory HLH in adults (Wang et al., 2015). Among 63 patients with refractory HLH, 29 had lymphoma-associated HLH, 22 had EBV-associated HLH, eight had an unknown cause, and four had primary HLH. During the evaluation after the 2- and 4-week treatment, 48 of 63 patients with HLH achieved treatment response (76.2%, ORR), 17 (27.0%) achieved CR, and 31 (49.2%) achieved PR. The study results showed that the DEP regimen had a high response rate in adult refractory HLH, confirming for the first time that the DEP regimen was an effective salvage treatment for adult refractory HLH. Compared to the classic HLH-94/HLH-04 regimen, this DEP regimen added a new drug, liposomal doxorubicin, while removing cyclosporine and replacing dexamethasone with methylprednisolone. The specific mechanism of action of liposomal doxorubicin in the treatment of HLH remains unclear. One possible mechanism is that after doxorubicin liposomes enter the human body, the liposomes can promote the enrichment of the lymphatic system and the accumulation of the drug in capillary permeability-increased sites (Huwyler et al., 2008; Schiffelers et al., 2001). This not only prolongs the half-life of the drug and extends the duration of action, but also more effectively inhibits the secretion of inflammatory cytokines in lymphocytes and the invasive damage of inflammatory factors to the body. However, experiments *in vivo* and vitro are still needed to further confirm its specific mechanism of action.

Subsequently, Wang et al. reported that based on the DEP regimen, PEG-aspargase (L-DEP regimen) was utilized to rescue and treat adult EBV-related HLH, and the results showed that the CR rate after treatment was 32.1%, the PR rate was 53.6%, and the ORR was 85.7% (Wang et al., 2016). They found that the EBV-DNA load in patients was significantly reduced after treatment with the L-DEP regimen, and that a high EBV-DNA load was an important factor in the poor prognosis of EBV-HLH. Wang et al. speculated that the main reason for this was that PEG-asparagine could target EBV-infected T and NK cells and induce L-asparagine hydrolysis after entering these cells, eventually inhibiting cell proliferation and leading to EBV-DNA decline. Lai et al. demonstrated the efficacy of L-DEP salvage therapy in adults and adolescents with relapsed refractory EBV-associated HLH (Lai et al., 2018). In addition to confirming the salvage therapeutic efficacy of L-DEP in adults with EBV-associated HLH, Zhao et al. confirmed the effectiveness of this regimen in children with recurrent and refractory EBV-associated HLH (Zhao et al., 2020). DEP is the basic salvage therapy for relapsed and refractory HLH. In addition to L-DEP combined with PEG-asparaginase, DEP combined with ruxolitinib (Ru-DEP regimen) has also been reported. HLH is an inflammatory disease characterized by an inflammatory cytokine storm as its main feature. The JAK-STAT pathway is important in the pathogenesis of inflammatory diseases and is involved in the pathogenesis and progression of HLH (Keenan et al., 2021). Ruxolitinib, a JAK 1/2 inhibitor, has been proven effective in blocking the validation pathway for the treatment of HLH (Keenan et al., 2021; Zhang et al., 2022; Zhou et al., 2025). The efficacy of ruxolitinib has been demonstrated in both mouse models and in patients with HLH (Zhang et al., 2022; Zhou et al., 2025; Keenan et al., 2024; Albeituni et al., 2019). Wang et al. reported a multicenter study on the salvage treatment of patients with relapsed and refractory HLH using the Ru-DEP protocol, in which the causes of HLH included EBV, primary HLH, MAS, lymphoma, drug, and unknown causes, and the reported total effective rate was 78% after a 2-week treatment (Wang et al., 2021). Furthermore, Wei et al. reported that a Ru-DEP regimen with or without PEG-asparagine (Ru-DEP ± L) was used to treat children with relapsed/refractory HLH (Wei et al., 2022). The results revealed that the ORR after treatment was 62.5%, and a good therapeutic effect was achieved. The Ru-DEP regimen exhibited good therapeutic effects. Furthermore, DEP-based rescue treatment of HLH with other drugs is being explored.

### DEP regimen for induction treatment of HLH

The DEP regimen exhibited optimal therapeutic effects in the salvage treatment of HLH and satisfactory therapeutic effects in the first-line induction treatment of HLH. For example, in a clinical study on HLH secondary to rheumatic disease, the 2-week ORR of the DEP regimen for newly diagnosed and relapsed/refractory patients was as high as 82.8% (Yin et al., 2024). In another study on lymphoma-associated HLH, DEP was significantly more effective as a first-line treatment than HLH-94, with an ORR of 89.4% and 68.0%, respectively, after 4 weeks of treatment (Meng et al., 2021). This study suggested that the main reason may be that liposomal doxorubicin in the DEP regimen can not only treat HLH but also lymphoma itself as an important drug in chemotherapy regimens for lymphoma. Furthermore, Pi et al. found in a multicenter study that a modified DEP regimen showed better therapeutic efficacy in the induction therapy for lymphoma-associated HLH (Pi et al., 2023). Additionally, DEP-based regimens have shown good therapeutic effects in the research reports of other types of HLH related to non-Hodgkin lymphoma (Meng et al., 2022; He et al., 2023; Zou et al., 2023). Moreover, as a first-line induction therapy, the L-DEP regimen showed good therapeutic efficacy in EBV-associated HLH (Chen et al., 2022). Presently, DEP-based regimens have attained good efficacy in first-line induction therapy for HLH; however, current studies have mainly focused on lymphoma and EBV-related HLH, and a large number of studies are still warranted to further confirm the therapeutic efficacy of DEP regimens.

### Improvement and limitation of the DEP regimen

The DEP regimen is constantly being optimized and improved. In terms of drugs, L-DEP regimens with increased PEG-asparaginase have been demonstrated to reduce the EBV-DNA load and are therefore recommended for EBV-associated HLH or NK-T cell lymphoma-associated HLH. In addition, the DEP regimen combined with ruxolitinib (Ru-DEP) achieved better therapeutic efficacy in the salvage treatment of HLH. In terms of the drug dose and cycle, etoposide was adjusted from the initial weekly dose to the current biweekly dose, methylprednisolone was reduced from the initial 15 mg/kg/d to 2 mg/kg/d, and the total treatment cycle was repeated from the initial 3 weeks to once every 2 weeks. Continuous improvements in the DEP regimen play an important role in improving the treatment response rate and reducing side effects. However, DEP has limitations. First, compared to HLH-94 and HLH-04, DEP adds liposomal adriamycin, a potent cytotoxic drug, whose use is associated with unavoidable systemic toxicity. Secondly, the specific mechanism of action of liposomal adriamycin in the treatment of HLH remains uncertain, and further study of this mechanism is warranted.

## Conclusion

In conclusion, through a review of current reports on the treatment of HLH using DEP regimens, it was found that the DEP regimen showed a better therapeutic effect not only as a salvage therapy regimen but also as a first-line induction therapy regimen Table 1. Moreover, DEP regimens are constantly being improved and have evolved to achieve better treatment results with fewer side effects. It is believed that DEP regimen will play an increasingly important role in the treatment of HLH.

**TABLE 1:** DEP regimens and responses for HLH.

References	Treatment opportunity	The cause of HLH	Number	Regimen	Time of response	CR	PR	ORR
Wang et al. (2014)	Salvage therapy	EBV; Lymphoma; FHLH; unknown cause	41	DEP	2 and 4 weeks	12 (29.3%)	20 (48.8%)	32 (78.1%)
Wang et al. (2015)	Salvage therapy	Lymphoma; EBV; FHLH; unknown cause	63	DEP	2 and 4 weeks	17 (27%)	31 (49.2%)	48 (76.2%)
Wang et al. (2016)	Salvage therapy	EBV	28	L-DEP	2 and 4 weeks	9 (32.1%)	15 (53.6%)	24 (85.7%)
Lai et al. (2018)	Initial treatment	EBV	6(adults and adolescents)	L-DEP	2 weeks	3 (50%)	2 (33.3%)	5 (83.3%)
Lai et al. (2018)	Salvage therapy	EBV	69(adults and adolescents)	L-DEP/DEP	2 weeks	18 (26.1%)	37 (53.6%)	55 (79.7%)
Zhao et al. (2020)	Salvage therapy	EBV	26(children)	L-DEP	2 and 4 weeks	5 (19.2%)	11 (42.3%)	16 (61.5%)
Wang et al. (2021)	Salvage therapy	EBV; FHLH; MAS; Lymphoma; drug; unknown cause	41	Ru-DEP	2 weeks	8 (19.5%)	24 (58.5%)	32 (78.0%)
Meng et al. (2021)	Induction therapy	Lymphoma	47	DEP	4 weeks	15 (31.9%)	27 (57.4%)	42 (89.4%)
Meng et al. (2022)	Induction therapy	NK/T-Lymphoma	14	L-DEP/DEP	2 weeks	0	11 (78.6%)	11 (78.6%)
Pi et al. (2023)	Induction therapy	Lymphoma	28	modifed DEP	4 weeks	8 (28.6%)	17 (60.7%)	25 (89.3%)
Chen et al. (2022)	Induction therapy	EBV	47	L-DEP	2 weeks	12 (25.5%)	26 (55.3%)	38 (80.9%)
Wei et al. (2022)	Salvage therapy	EBV; FHLH; unknown cause	16	RuDEP+/-L	2 weeks	3 (18.8%)	7 (43.8%)	10 (62.5%)
Zou et al. (2023)	Induction therapy	Lymphoma	82	DEP/L-DEP	2 weeks	5(6.1%)	55 (67.1%)	22 (26.8%)
He et al. (2023)	Induction therapy	NK/T-Lymphoma	28	DEP	2 weeks	0	19 (67.9%)	19 (67.9%)
Yin et al. (2024)	Induction/salvage therapy	Rheumatic disease	58	DEP	2 weeks	8 (13.8%)	40 (69%)	48 (82.8%)

DEP, liposomal doxorubicin combination with etoposide and methylprednisolone; HLH, hemophagocytic lymphohistiocytosis; L PEG-aspargase; CR, complete response; PR, partial response; ORR, overall response rate; EBV, Epstein-Barr virus; FHLH, familial hemophagocytic lymphohistiocytosis; MAS macrophage activation syndrome, Ru ruxolitinib.
